# Cardioprotective Effects of 20(S)-Ginsenoside Rh2 against Doxorubicin-Induced Cardiotoxicity *In Vitro* and *In Vivo*

**DOI:** 10.1155/2012/506214

**Published:** 2012-10-17

**Authors:** Hongbo Wang, Pengfei Yu, Haitao Gou, Jianqiao Zhang, Mei Zhu, Zhen-hua Wang, Jing-wei Tian, Yong-tao Jiang, Feng-hua Fu

**Affiliations:** ^1^Department of Pharmacology, School of Pharmacy, Yantai University, Yantai 264005, China; ^2^State Key Laboratory of Long-Acting and Targeting Drug Delivery Technologies, Luye Pharma Group Ltd., Yantai 264003, China

## Abstract

Doxorubicin (DOX) is considered as one of the best antineoplastic agents. However, its clinical use is restricted by its associated cardiotoxicity, which is mediated by the production of reactive oxygen species. In this study, 20(S)-ginsenoside Rh2 (Rh2) was explored whether it had protective effects against DOX-induced cardiotoxicity. *In vitro* study on H9C2 cell line, as well as *in vivo* investigation in one mouse and one rat model of DOX-induced cardiomyopathy, was carried out. The results showed that pretreatment with Rh2 significantly increased the viability of DOX-injured H9C2 cells. In the mouse model, Rh2 could suppress the DOX-induced release of the cardiac enzymes into serum and improved the occurred pathological changes through ameliorating the decreased antioxidant biomolecules and the cumulated lipid peroxidation malondialdehyde in heart tissues. In the rat model, Rh2 could attenuate the change of ECG resulting from DOX administration. Furthermore, Rh2 enhanced the antitumor activity of DOX in A549 cells. Our findings thus demonstrated that Rh2 pretreatment could effectively alleviate heart injury induced by DOX, and Rh2 might act as a novel protective agent in the clinical usefulness of DOX.

## 1. Introduction

Doxorubicin (DOX), one anthracycline drug, has been proved to be one of the most active antitumor drugs developed to date for the treatment of malignant neoplasias, such as solid tumors, leukemia, and lymphoma [1]. However, its clinical usefulness is restricted to its cardiotoxicity, which might be either acute or chronic and eventually lead to cardiomyopathy following severe heart failure [2]. The efficacy of DOX, therefore, is severely confined by the cumulative dose-dependent cardiotoxicity and the emerging drug resistance [1, 3]. Thus, new compounds, which can reduce the severe side effect while maintaining and/or enhancing its antitumor activity, will benefit the success of its use in clinic. 

Considerable research has focused on elucidating the mechanisms of cardiomyopathy induced by DOX as well as on finding reasonable ways to prevent its development and progression [3, 4]. To date, the exact molecular mechanism of DOX-induced cardiotoxicity still remains unclear. Among the proposed hypothesis, the generation of reactive oxygen species (ROS) induced by DOX is one center mediator of direct and indirect cardiac adverse consequences [1, 2, 5]. It has been well demonstrated that DOX catalyzed the reduction of oxygen by nicotinamide adenine dinucleotide phosphate (NADPH) to form a superoxide radical, which subsequently caused membrane lipid peroxidation and enzyme inactivation [4, 6]. Besides, the intrinsic apoptosis, which occurred in a p53-dependent manner in cardiomyocytes [7], has been found to be involved in the development and progress of DOX-induced cardiomyopathy [8]. Available lab evidences also confirmed that antioxidants, such as dexrazoxane [9], thrombopoietin [8], schisandrin B [3], probucol [10], and davallialactone [11], could display protective effect against the DOX-induced cardiotoxicity both *in vitro* and *in vivo*. Therefore, novel antioxidants might be a potential candidate for the prevention and treatment of DOX-induced cardiomyopathy. 

**Figure 1:**
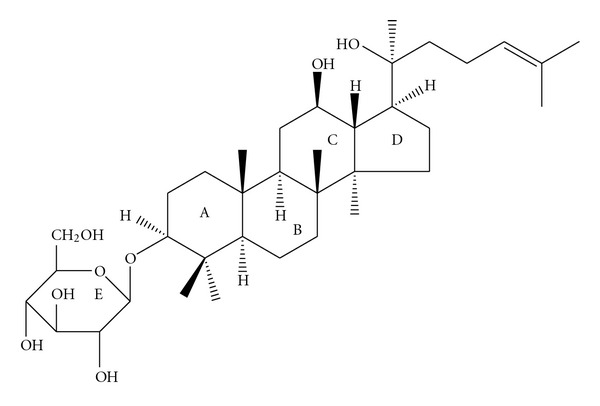
Chemical structure of ginsenoside Rh2.

Ginseng has been prescribed as one herbal medication for over 2000 years and has demonstrated lots of direct or indirect cardiovascular benefits, such as antihypertensive effects and attenuation of heart failure [12]. Experiments have shown that all of the therapeutic effects were attributed primarily to the presence of different ginsenosides, which bestowed these ginsengs with different pharmacodynamic profiles [12, 13]. Among all the reported ginsenosides, Re, Rb, and Rg1 have shown the most promising evidences for the protection against cardiac injury *in vitro* and *in vivo *[14–16]. Ginsenoside Rh2 (Figure 1), first isolated from red ginseng in 1983, is a trace active ingredient of ginseng and has the molecular formula C_36_H_62_O_8_ [17, 18]. In the early stage of investigations, Rh2 displayed marked anticancer activity via inhibiting cell growth and inducing apoptosis in several cancer cells; at the same time, Rh2 showed quite low toxicity toward normal cells [18, 19]. Recently, Rh2was reported to potently protect the ischemia-reperfusion brain injury in rat and inhibit prostaglandin-E2 synthesis in lipopolysaccharide-stimulated RAW264.7 cells [20]. We propose that Rh2 could protect against the cardiomyopathy induced by DOX. In this study, we perform *in vitro* and *in vivo* strategies to explore the effect of Rh2 on the DOX-induced cardiotoxicity. 

## 2. Materials and Methods

### 2.1. Materials

Ginsenoside Rh1, Rh2, Re, Rg1,Ocotillol,RT5, and F11 were all isolated by Shandong Luye Pharmaceutical Company (Yantai, China) and obtained as white powder. Purity of the compounds used in the present study was all checked by HPLC and found to be higher than 99%. *In vitro*, all the ginsenosides and Doxorubicin (Zhejiang Hisun Pharmaceutical Co, Ltd. China) were dissolved in DMSO and stored in −20°C for less than 1 month before use. The vehicle (DMSO) was used as a control in all experiments at a maximum concentration of 0.1%. *In vivo* Rh2 and DOX were dissolved in 1% carboxymethylcellulose sodium (CMCS) and 0.9% sodium chloride as proposed dose, respectively.

### 2.2. Cell Lines and Cell Culture

The rat cardiomyoblast cell line H9C2 and human lung cancer cell line A549 were purchased from Cell Culture Center of Institute of Basic Medical Sciences, Chinese Academy of Medical Sciences. All the cells were cultured in DMEM media supplemented with 10% fetal calf serum, penicillin (100 U/mL), and streptomycin (10 *μ*g/mL) (Gibco BRL, NY, USA) and incubated at 37°C in a humidified air atmosphere containing 5% CO_2_. All the cells were harvested in their exponentially growing phase.

### 2.3. Cell Proliferation Assays

The viability of the cells was evaluated using MTT assay as reported previously [21]. Briefly, cells were seeded into 96-well plates and then treated with the tested articles as the desired concentration for 24 h. MTT solution was added into the wells and incubated for 2 h. After the medium was removed, DMSO was added into each well. The plates were gently agitated until the color reaction was uniform and the OD_570_ was determined using a microplate reader (Wellscan MK3, Labsystems Dragon). 

### 2.4. Animals

Male Swiss mice (18–22 g) and male Sprague-Dawley rats (190–220 g) were obtained from Shandong Luye Pharmaceutical Company (Yantai, China). The animals were housed in a light and temperature-controlled room (21-22°C, humidity 50–65%) and kept on a standard diet and water. All of the experiments were performed in accordance with the Guideline for Care and Use of Experimental Animals of Experimental Animal Research Committee of Yantai University.

### 2.5. *In Vivo* Mouse Model of DOX-Induced Cardiotoxicity

Male Swiss mice were randomly divided into 5 groups (*n* = 10). The control group was given total 6 doses of 0.9% NaCl intraperitoneal injection (i.p.) every other day and 8 doses of CMC gavage (P.O.) daily. The DOX group was administrated total 6 doses of DOX dissolved in 0.9% NaCl i.p. at 3 mg/kg (accumulative dose 18 mg/kg) every other day and total 8 doses of CMC P.O. daily. The pretreated groups received total 8 doses of Rh2 at 5 mg/kg, 10 mg/kg, and 20 mg/kg daily with the first administration 24 h before DOX injection. 

The blood sampling and tissue preparation were performed under anesthesia with ketamine and xylazine 24 h after last dose. Serum was prepared by centrifuge, and the creatine kinase (CK), aspartate transaminase (AST), and lactate dehydrogenase (LDH) were detected by the commercial kits (Nanjing, China) following the instruction. After the mice were sacrificed, the hearts were removed quickly. Half of tissues were cooled, homogenized, and then determined for superoxide dismutase (SOD), malondialdehyde (MDA), glutathione (GSH), and catalase (CAT) with commercial kits (Nanjing, China). The other parts of heart tissue were carried on for the histological examination, in which all specimens were analyzed and pictures taken by two pathologists with blind investigation.

### 2.6. *In Vivo* Rat Model for ECG Examination

Sprague-Dawley rats were randomly divided into 6 groups (*n* = 6 in each group). The control group was given total 4 doses of 0.9% NaCl i.p. every other day and total 8 doses of CMC P.O. The DOX group was administrated total 4 doses of DOX dissolved in 0.9% NaCl i.p. at 2 mg/kg (accumulative dose 8 mg/kg) every other day, and the Rh2 group only received total 8 doses of Rh2 daily via gavage at 20 mg/kg. The pretreated groups received total 8 doses of Rh2 at 5 mg/kg, 10 mg/kg, and 20 mg/kg daily with the first administration 24 h before the first of 4 doses of DOX. 

The treated Sprague-Dawley rats were anesthetized with ketamine and xylazine 24 h after last administration, and then the ECG were recorded by electrocardiograph (ECG-901A, China) following the instruction.

### 2.7. Data Analysis and Statistics

The results are presented as mean ± SD. Comparisons between more than 2 groups were performed by analysis of variance (one-way ANOVA); then Student *t*-test was performed.* P* ≤ 0.05 was used as the level of statistical significance unless indicated otherwise.

## 3. Results

### 3.1. Rh2 Increased the Viability of DOX-Treated H9C2 Cells

**Figure 2:**
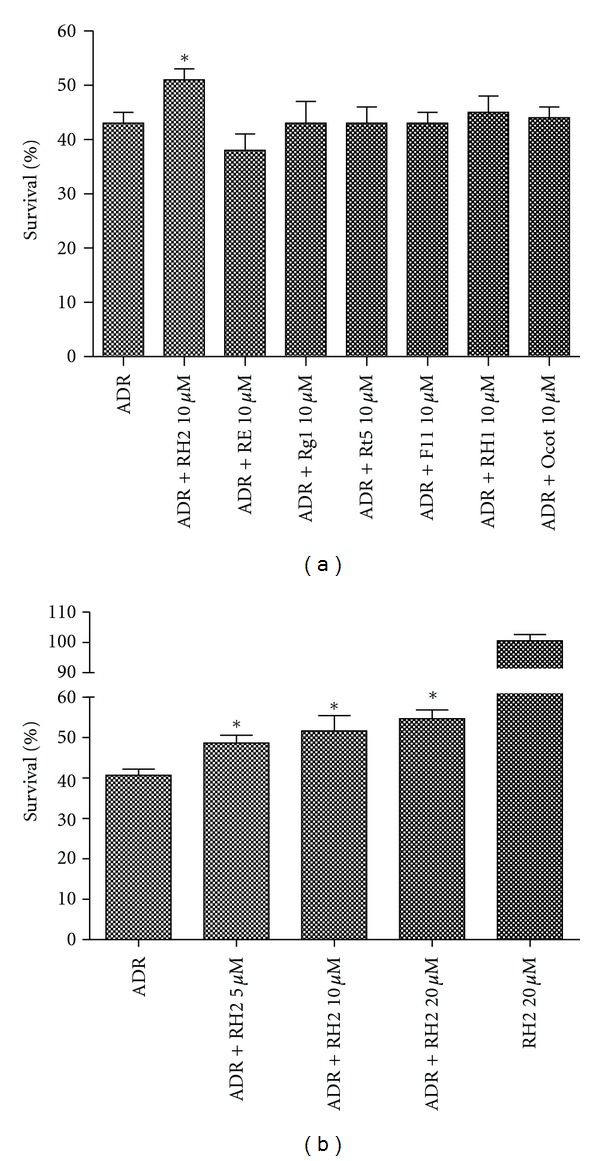
Effect of Rh2 on the cytotoxic activity of DOX *in vitro*. H9C2 cells were seeding into 96-well plate and treated as indicated. The cell viability was detected by MTT assay after 24 h incubation. All data are expressed as means ± SD (*n* = 3). *P < 0.05, compared with DOX group.

MTT assay was performed to explore the effect of seven ginsenosides against the cardiotoxic effect of DOX in H9C2 cells. DOX at the concentration of 2 *μ*M was found to significantly inhibit the growth of H9C2 cells (Figure 2) after 24 h incubation. Among all the tested ginsenosides at one concentration of 10 *μ*M, Rh2 was the most powerful compound to attenuate the decrease in the viability of H9C2 cells after DOX treatment (Figure 2). Then, different concentrations of Rh2 were further to be tested using the same condition. Rh2 at the concentration of 5 *μ*M, 10 *μ*M, and 20 *μ*M, which were preadministrated 2 h before DOX exposure, was shown to significantly attenuate the cytotoxic effect of DOX with increasing the survival rate in a dose-dependent manner (Figure 2). At the same time, Rh2 alone had no effect on the cell growth. 

### 3.2. Rh2 Protected against DOX-Induced Cardiotoxicity in Mouse Models

#### 3.2.1. Serum CK, LDH, and AST

**Figure 3:**
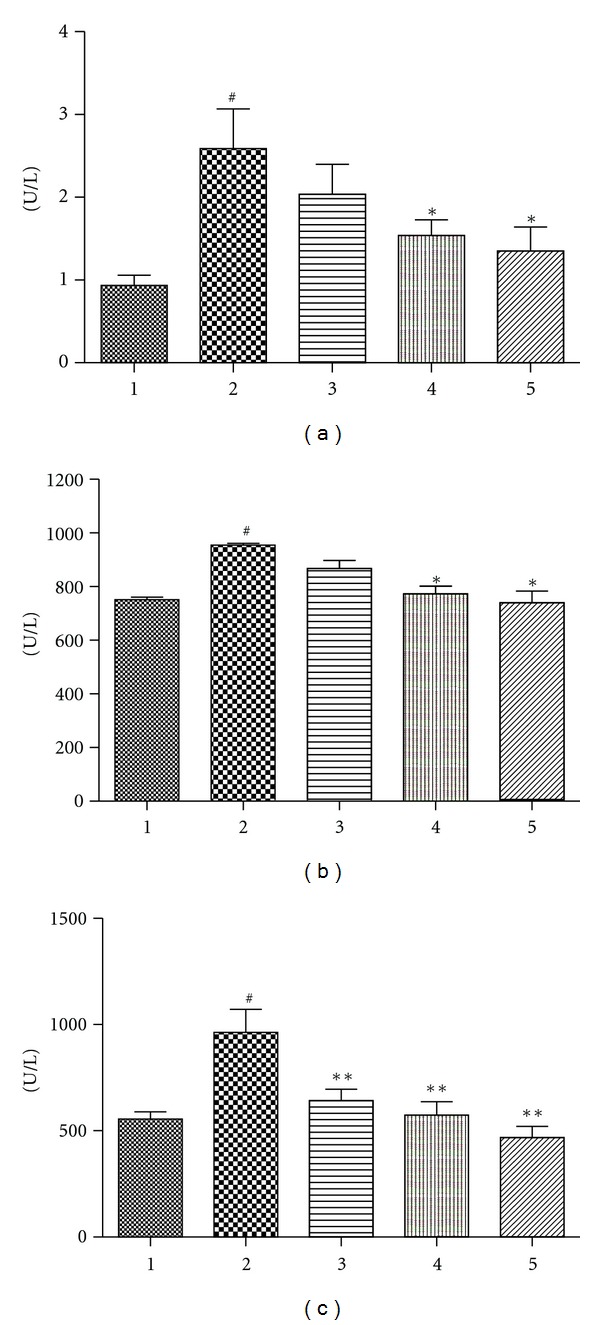
Effect of Rh2 on serum CK, LDH, and AST in DOX-treated animals. Animal serum was prepared, and then the serum CK (a), LDH (b), and AST (c) were detected. 1: Control group; 2: DOX group; 3: DOX plus Rh2 5 mg/kg group; 4: DOX plus Rh2 10 mg/kg group; 5: DOX plus Rh2 20 mg/kg group. All data are expressed as means ± SD (*n* = 10). ^#^ *P* < 0.01, compared with control group; *P < 0.05, compared with DOX group; **P < 0.01, compared with DOX group.

The CK, LDH, and AST in serum are the biomarkers of heart tissue damage [22]. As cardiotoxic agents, DOX did significantly increase the level of CK, LDH, and AST in the treated animals (Figure 3; *P* < 0.01, compared with control group), which indicated the occurrence of heart tissue injury. Compared with DOX treated group, however, the animals pretreated with different dose of Rh2 were found to have an obvious reduction in AST (*P* < 0.01, compared with DOX group). Rh2 at dose of 10 mg/kg and 20 mg/kg was observed to significantly decrease the elevated CK and LDH (*P* < 0.05, compared with DOX group). 

#### 3.2.2. Tissue SOD, CAT, GSH, and MDA

**Figure 4:**
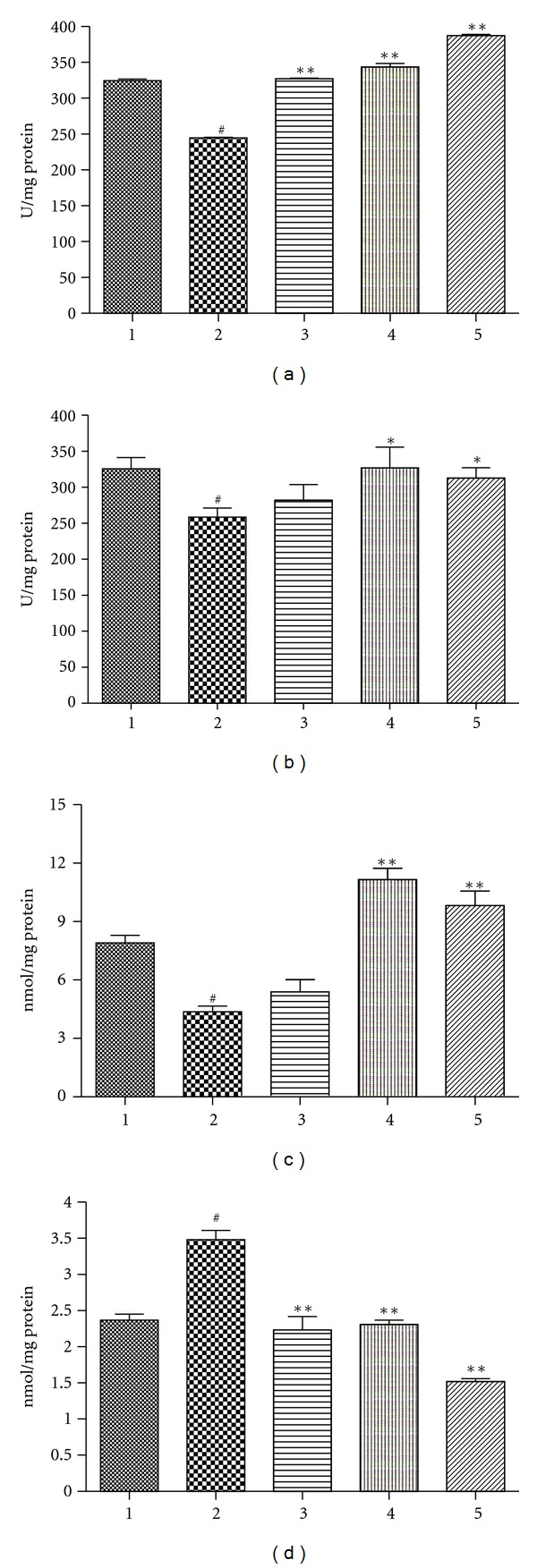
Effect of Rh2 on the content of anti-oxidant biomolecules and the content of MDA in heart tissue. The tissues were cooled, homogenized, and then determined for SOD (a), CAT (b), GSH (c), and MDA (d). Groups were the same as in Figure 3. All data are expressed as means ± SD (*n* = 10). ^#^ *P* < 0.05, compared with control group; *P < 0.05, compared with DOX group; **P < 0.01, compared with DOX group.

SOD, GSH, and CAT are important antioxidant biomolecules in the tissue against oxidant stress, especially in heart tissue. After treatment with DOX, the SOD, GSH, and CAT in mouse heart tissue were significantly decreased (Figure 4; *P* < 0.05, compared with the control group). As a result of reduction of the anti-oxidants, the MDA was found to be significantly elevated. Rh2, with which the animals were pretreated with different dosage, significantly alleviated the reduction of the SOD, GSH, and CAT and decreased the content of MDA. 

#### 3.2.3. Histological Examination

**Figure 5:**
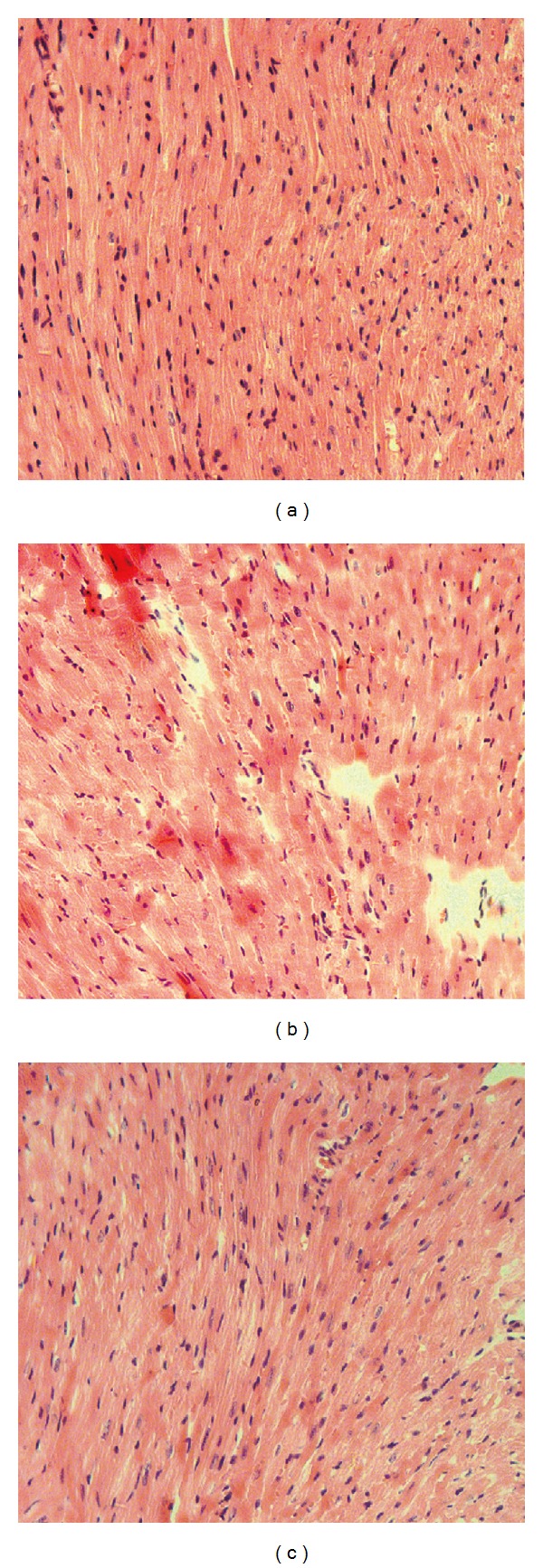
Effect of Rh2 on the histopathological changes in left ventricles. Half heart was firstly fixed and followed with HE staining. Light microscope observations were carried out, and the representative fields were shown. (a) Control group; (b) DOX group; (c) DOX plus Rh2 20 mg/kg group (400x magnifications).

Light microscope observations of the left ventricles were carried out. The animals in control groups were observed with normal morphology of cardiomyocytes. In animals treated with DOX, however, loss of cross-striation, congestive edema of cardiac muscle interstitial, myocardialendochylemapuffing and sarcoplasmic matrix partly resorbed, myocardial cells derangement, enhanced acidophilia, granular degeneration, and cytoplasmic vacuolization were clearly observed (Figure 5). In mice pretreated with different dosage of Rh2, less histopathological changes were observed.

### 3.3. Rh2 Protected against DOX-Induced Cardiotoxicity in Rat Models

**Figure 6:**
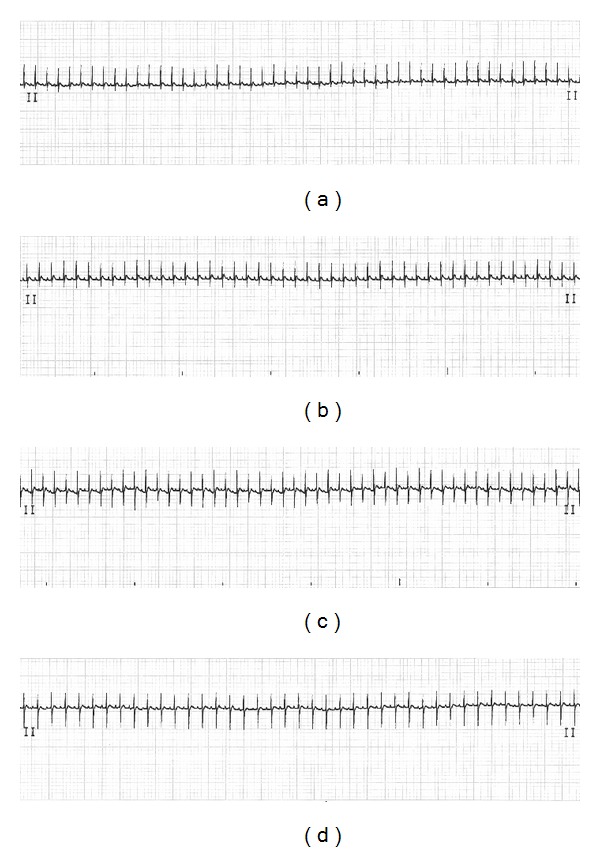
Effect of Rh2 on the cardiac function parameters in ECG assay. Animals were anesthetized 24 h after last administration, and then the ECG were recorded by electrocardiograph. The representative pictures were shown. (a) Control group; (b) Rh2 20 mg/kg group; (c) DOX group; (d) DOX plus Rh2 20 mg/kg group.

The ECG recorders were carried out 24 h after the last administration. The animals with DOX injections were manifested with elevated heart rates and widened QRS complex (Table 1), which indicated that DOX-treated rats suffered from cardiomyopathy. However, the rats with Rh2 preadministration were recorded with relatively normal ECG features (Figure 6). Consistently, Rh2 treatment alone had no effect. 

### 3.4. Rh2 Enhanced the Antitumor Activity of DOX in A549 Cells

**Figure 7:**
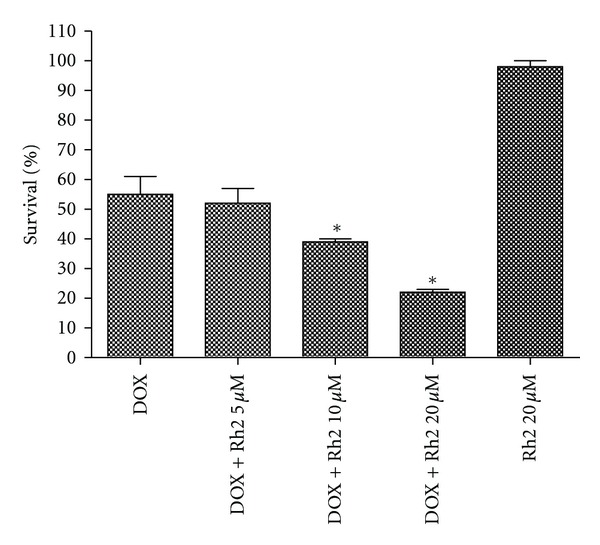
Effect of Rh2 on the antitumor activity of DOX *in vitro.* A549 were seeded and treated with the same condition mentioned in Figure 2. All data were expressed as means ± SD (*n* = 3). *P < 0.05, compared with DOX group.

The effect of Rh2 on the antitumor activity of DOX was tested in A549, a human lung cancer cell line. Rh2, which was treated with the same condition as H9C2 cells mentioned above, could enhance the antitumor activity of DOX in a dose-dependent manner (Figure 7). However, Rh2 alone did not display any growth inhibition against these cancer cells at the tested concentration.

## 4. Discussion

The cardiomyopathy induced by repeated administration of DOX is frequent and devastating, and the adverse complications lead to morbidity and/or poor quality of life [1, 2]. To mitigate that, research focusing on finding “good agents” that do not compromise its antitumor activity and bestowed great preventive or protective profile against the cardiac dysfunction at same time is being conducted. In this study, we firstly demonstrated the cardioprotective effects of Rh2 against DOX-induced cardiotoxicity *in vitro* and *in vivo.*

Ginseng has already been proved to exert potential benefits on vascular function via its antioxidant properties [12], which might be used to eliminate the key mediators for the DOX-induced cardiotoxicity [23]. We first tested the effect of seven ginsenosides against the cytotoxic activity of DOX in H9C2 cell, which was the most popular cell model used to screen protective agents [24]. Among all the tested compounds, Rh2 was the most effective agent to display the protective effect against the cytotoxic effect of DOX (Figure 2). No obviously protective activities were observed in the left six ginsenosides, which were not fully consistent with previous literatures [14–16]. One possible reason is that they might exert cardiac protective activity in the different disease model through a direct or indirect way.

The content of CK, LDH, and AST in serum was biomarkers used as monitoring myocardia cells damage [22]. During the injury of cardiac myocyte, these special enzymes will leak into the serum, which can easily be detected from blood samples. Based on the published literature [8], the cumulative dose of DOX at 18 mg/kg was enough to induce the cardiomyopathy in mice. The serum CK, LDH, and AST were significantly increased in the DOX-treated animals (Figure 3). The increased serum enzymes were suppressed in the Rh2 pretreated animals, which indicated Rh2 could indeed attenuate the DOX-induced cardiac injury. Consistent with the *in vitro* and the serum enzyme data, the histological examination of the animal's heart tissue also showed that pretreatment of Rh2 could dramatically alleviate the histopathologic lesion induced by DOX (Figure 5). The protective activity of Rh2 was also observed in rat using the ECG recorder (Figure 6 and Table 1), which was the most useful method to evaluate heart function under noninvasive conditions. These findings demonstrated that the ginsenoside Rh2 did have cardioprotective activity against the DOX-induced cardiomyopathy both *in vitro* and* in vivo*.

Dexrazoxane, which worked as an anti-oxidant, was the one and only drug approved by the FDA to prevent and treat the DOX-induced cardiomyopathy [25]. In this study, Rh2 was demonstrated with the comparable protective activity, which was also similar to several published anti-oxidants, such as schisandrin B, davallialacton, and probucol. These compounds mentioned above were all reported to exert their cardioprotective activities through detoxification of free radicals [4, 6, 10], which contributed to the occurrence of DOX-induced pathology changes [1]. All of these evidences indicated the protective effect of Rh2 in H9C2 cell might contribute to its direct anti-oxidant property. 

The generation of ROS induced by DOX was the central cause of numerous direct and indirect cardiac adverse consequences [2]. Under current condition, the major anti-oxidant biomolecules, such as SOD, GSH, and CAT were decreased in heart tissue from animals treated with DOX. As a result, MDA, one byproduct of lipid peroxidation, was observed with dramatic increase. This lipid peroxidation could diminish enzyme activity by oxidizing the active site or by forming protein cross-links, which might aggravate the oxidant stress against the heart tissue [1]. Pretreatment with Rh2 could increase the SOD, GSH, and CAT, activity in the tested tissue; as a consequence of that, the MDA would be cleared by these anti-oxidant biomolecules. Thus, Rh2 could improve the “endogenous antioxidant reserve,” which has been suggested to improve myocardial structure (Figure 5) and cardiac function (Figure 6). The exact mechanism for the maintenance of endogenous antioxidants (SOD, GSH, and CAT) by Rh2, which was decreased by DOX treatment, is still not very clear. However, our data clearly showed that Rh2 could work as a protective agent against the cardiotoxic effects of DOX. 

Another question addressed was the effect of Rh2 on the antitumor activity of DOX. From the* in vitro *data, Rh2 did not restrain the antiproliferation of DOX in the human lung cancer cells, but enhanced its antitumor activity, which was totally different with its effects on H9C2 cells. One possible interpretation for this difference is that this occurs in a cell-dependent manner, in which the cell-selective characteristic will further benefit its coadministration in clinic [3]. This interesting finding, which was also observed in several published anti-oxidants [3, 11], supports the further investigation for Rh2 as a protector against DOX-induced cardiotoxicity. 

## 5. Conclusion

In summary, the present study firstly reported here a novel protective role of Rh2 against DOX-induced cardiomyopathy, which might be related to the role of Rh2 in the maintenance of endogenous anti-oxidants status. Our data thus provides the evidence for coadministration of Rh2 with doxorubicin to attenuate the latter's cardiotoxicity and synergize its antitumor activity.

## Figures and Tables

**Table 1:** Effects of doxorubicin and Rh2 on the parameters of ECG in tested rats (means ± SD, *n* = 6).

Group	Control	Rh2 20 mg/kg	DOX 2 mg/kg	DOX plus Rh2 5 mg/kg	DOX plus Rh2 10 mg/kg	DOX plus Rh2 20 mg/kg
Heart rate (times·min^−1^)	426.2 ± 31.6	401.2 ± 38.9	472 ± 31.9	450.6 ± 59.1	408.2 ± 39.4*	411.4 ± 50.1
QRS complex (sec)	0.056 ± 0.005	0.052 ± 0.008	0.075 ± 0.006^#^	0.06 ± 0.019	0.056 ± 0.011*	0.054 ± 0.005**

^#^ *P* < 0.01, compared with control group; *P < 0.05, compared with DOX group; **P < 0.01, compared with DOX group.

All data were expressed as means ± SD (*n* = 6).

## References

[1] Minotti G, Menna P, Salvatorelli E, Cairo G, Gianni L (2004). Anthracyclines: molecular advances and pharmacologie developments in antitumor activity and cardiotoxicity. *Pharmacological Reviews*.

[2] Scott JM, Khakoo A, Mackey JR, Haykowsky MJ, Douglas PS, Jones LW (2011). Modulation of anthracycline-induced cardiotoxicity by aerobic exercise in breast cancer: current evidence and underlying mechanisms. *Circulation*.

[3] Li L, Lu Q, Shen Y, Hu X (2006). Schisandrin B enhances doxorubicin-induced apoptosis of cancer cells but not normal cells. *Biochemical Pharmacology*.

[4] Arunachalam S, Kim SY, Lee SH (2012). Davallialactone protects against adriamycin-induced cardiotoxicity *in vitro* and *in vivo*. *Journal of Natural Medicines*.

[5] Olson RD, Mushlin PS, Brenner DE (1988). Doxorubicin cardiotoxicity may be caused by its metabolite, doxorubicinol. *Proceedings of the National Academy of Sciences of the United States of America*.

[6] Li N, Pan Q, Han W, Liu Z, Hu X (2007). Schisandrin B prevents doxorubicin-induced cardiotoxicity via enhancing glutathione redox cycling. *Clinical Cancer Research*.

[7] Zhu W, Soonpaa MH, Chen H (2009). Acute doxorubicin cardiotoxicity is associated with p53-induced inhibition of the mammalian target of rapamycin pathway. *Circulation*.

[8] Li K, Sung RYT, Wei ZH (2006). Thrombopoietin protects against *in vitro* and *in vivo* cardiotoxicity induced by doxorubicin. *Circulation*.

[9] Imondi AR, Della Torre P, Mazué G (1996). Dose-response relationship of dexrazoxane for prevention of doxorubicin- induced cardiotoxicity in mice, rats, and dogs. *Cancer Research*.

[10] Siveski-Iliskovic N, Kaul N, Singal PK (1994). Probucol promotes endogenous antioxidants and provides protection against adriamycin-induced cardiomyopathy in rats. *Circulation*.

[11] Xiao J, Sun GB, Sun B (2012). Kaempferol protects against doxorubicin-induced cardiotoxicity *in vivo* and *in vitro*. *Toxicology*.

[12] Karmazyn M, Moey M, Gan XT (2011). Therapeutic potential of ginseng in the management of cardiovascular disorders. *Drugs*.

[13] Attele AS, Wu JA, Yuan CS (1999). Ginseng pharmacology: multiple constituents and multiple actions. *Biochemical Pharmacology*.

[14] Schibilsky D, Beyersdorf F, Goebel U (2010). Amelioration of rat cardiac cold ischemia/reperfusion injury with inhaled hydrogen or carbon monoxide, or both. *Journal of Heart and Lung Transplantation*.

[15] Kupriyanov VV, Xiang B, Sun J, Jilkina O (2002). The effects of drugs modulating K+ transport on Rb+ uptake and distribution in pig hearts following regional ischemia: 87Rb MRI study. *NMR in Biomedicine*.

[16] Zhu D, Wu L, Li CR (2009). Ginsenoside Rg1 protects rat cardiomyocyte from hypoxia/reoxygenation oxidative injury via antioxidant and intracellular calcium homeostasis. *Journal of Cellular Biochemistry*.

[17] Zhang J, Zhou F, Wu X (2012). Cellular pharmacokinetic mechanisms of adriamycin resistance and its modulation by 20(S)-ginsenoside Rh2 in MCF-7/Adr cells. *British Journal of Pharmacology*.

[18] Li B, Zhao J, Wang CZ (2011). Ginsenoside Rh2 induces apoptosis and paraptosis-like cell death in colorectal cancer cells through activation of p53. *Cancer Letters*.

[19] Park EK, Lee EJ, Lee SH (2010). Induction of apoptosis by the ginsenoside Rh2 by internalization of lipid rafts and caveolae and inactivation of Akt. *British Journal of Pharmacology*.

[20] Park EK, Choo MK, Oh JK, Ryu JH, Kim DH (2004). Ginsenoside Rh2 reduces ischemic brain injury in rats. *Biological and Pharmaceutical Bulletin*.

[21] Wang H, Li H, Zuo M (2008). Lx2-32c, a novel taxane and its antitumor activities *in vitro* and *in vivo*. *Cancer Letters*.

[22] Rajadurai M, Stanely Mainzen Prince P (2007). Preventive effect of naringin on cardiac markers, electrocardiographic patterns and lysosomal hydrolases in normal and isoproterenol-induced myocardial infarction in Wistar rats. *Toxicology*.

[23] Sun X, Zhou Z, Kang YJ (2001). Attenuation of doxorubicin chronic toxicity in metallothionein-overexpressing transgenic mouse heart. *Cancer Research*.

[24] Shi R, Huang CC, Aronstam RS, Ercal N, Martin A, Huang YW (2009). N-acetylcysteine amide decreases oxidative stress but not cell death induced by doxorubicin in H9c2 cardiomyocytes. *BMC Pharmacology*.

[25] Hellmann K (1998). Overview and historical development of dexrazoxane. *Seminars in Oncology*.

